# Antinuclear antibodies as a risk factor for ischemic stroke or death in elderly patients with atrial fibrillation despite anticoagulation

**DOI:** 10.1007/s10067-025-07638-y

**Published:** 2025-08-22

**Authors:** Maksymilian Hanarz, Michał Ząbczyk, Joanna Natorska, Elżbieta Pociask, Anetta Undas

**Affiliations:** 1 Department of Thromboembolic Disorders, Institute of Cardiology, Jagiellonian University Medical College, 80 Pradnicka St., 31-202 Kraków, Poland; 2 Krakow Centre for Medical Research and Technologies, St. John Paul II, Hospital, Krakow, Poland; 3 Department of Biocybernetics and Biomedical Engineering, AGH University of Science and Technology, Krakow, Poland

**Keywords:** Antinuclear antibodies, Atrial fibrillation, Death, Ischemic stroke

## Abstract

**Objective:**

Antinuclear antibodies (ANA) at low titers of 1:40 may be present in up to 30% of healthy individuals and have been associated with increased cardiovascular mortality, but their role in atrial fibrillation (AF) remains unclear. This study assessed ANA prevalence and its association with outcomes in AF patients.

**Methods:**

In a cohort of 240 AF patients on anticoagulant therapy (median age 69, median CHA2DS2-VASc = 4), without any ANA-related autoimmune diseases, we determined ANA (positive if > 20 ELISA units [EU]) along with antiphospholipid antibodies in accordance to ACR/EULAR (ACR/EULAR-aPL). During a median follow-up of 52 months, ischemic stroke (IS), transient ischemic attack (TIA), cardiovascular (CV) death, major bleeding, and a composite endpoint (defined as IS, TIA, or CV death) were recorded.

**Results:**

43 patients (17.9%) were positive for ANA (mean 22.9 EU), including 20 (46.5%) with positive ACR/EULAR-aPL. ANA-positive patients were older (by 9.9 years), predominantly female (65.1%), and had higher CHA2DS2-VASc scores (median 5 vs 4; all *P* < 0.001). IS or CV death occurred in 30 patients (12.5%, 3% per year) who were ANA positive. ANA positivity was associated with the composite endpoint occurrence (odds ratio [OR] = 2.84, 95% confidence interval [CI] 1.21–6.65). Furthermore, the presence of both ANA and ACR/EULAR-aPL positivity significantly increased the likelihood of the composite endpoint (OR = 4.85, 95%CI 1.75–13.43).

**Conclusions:**

Positive ANA coexisting with ACR/EULAR-aPL may contribute to the failure of oral anticoagulation in AF patients, highlighting a potential role of autoimmune mechanisms in thromboembolism associated with this common arrhythmia.

**Table:** 

**Key Points** • *The role of antinuclear antibodies (ANA) in atrial fibrillation (AF) remains underexplored, despite their known association all-cause mortality*. • *This study addresses the gap by evaluating coexistence of ANA and antiphospholipid antibodies in accordance to ACR/EULAR (ACR/EULAR-aPL) in anticoagulated AF patients without autoimmune disease*. • *ANA positivity, particularly when coexisting with ACR/EULAR-aPL, was associated with an increased risk of thromboembolic events despite anticoagulation*. • *ANA was more frequently detected in patients who experienced the composite endpoint of ischaemic stroke, transient ischaemic attack, or cardiovascular death*.

**Graphical abstract:**

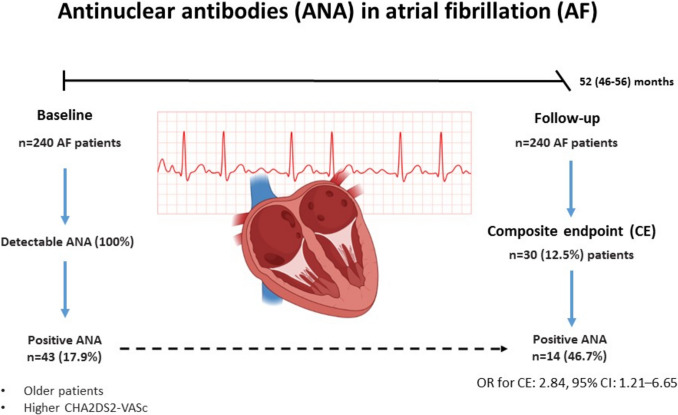

## Introduction

Atrial fibrillation (AF) is the most common cardiac arrhythmia, with increasing impact on global morbidity and mortality in aging populations, primarily due to its association with ischemic stroke (IS) [1–3]. Although well-established risk factors in scoring systems, e.g. CHA2DS2-VASc are primarily demographic and clinical, their limited effectiveness has spurred the search for novel factors and biomarkers to improve risk stratification in AF, including better identification of subjects more likely to experience thromboembolism despite oral anticoagulation [4]. In the general population, low-titer antinuclear antibodies (ANA) at a dilution of 1:40 may be present in up to 30% of healthy individuals [5, 6], with higher prevalence in females (over 9.04% more than males) [7] and increasing with age (2% per year) [8]. Notably, the presence of ANA in the general population has been shown to correlate with increased all-cause mortality and cardiovascular (CV) death with a hazard ratio (HR) of] 4.62 per 1 standard deviation increase in log-transformed ANA (95% confidence interval [CI]: 4.5–4.7; p < 0.001) for all-cause mortality and an HR of 1.42 (95% CI: 1.13–1.77; *P* = 0.002) for CV death [9, 10].

The impact of ANA on thromboembolic risk appears to be influenced by its specificity. For example, antibodies targeting double-stranded DNA (dsDNA) are strongly associated with autoimmune diseases and cardiovascular events, while non-specific ANA confer a lower but still noteworthy risk [11]. ANA are well-established markers of autoimmune disorders, detected in over 90% of patients with systemic sclerosis [12] and in approximately 24–60% of those with dermatomyositis [13]. ANA positivity is also observed in up to 83% of patients with primary Sjögren’s syndrome and as high as 99.8% in systemic lupus erythematosus (SLE) [14]. However, ANA can also be detected in the general population: up to 25% of individuals may test positive, although significantly elevated titers are observed in only about 2.5% [15]. For example ANA at a serum dilution of 1:100 or higher were more frequently detected in patients following venous thromboembolism (VTE) compared to age-matched healthy controls [11]. Data on ANA prevalence in AF patients are limited. Hu et al. [16] reported similar ANA levels (approximately 10.5 U/mL) in 71 non-valvular Chinese AF patients and 75 age- and gender-matched healthy controls, but the rate of positive ANA has not been reported. Importantly, ANA often coexists with antiphospholipid antibodies in accordance to ACR/EULAR (ACR/EULAR-aPL), especially in SLE patients [17]. Positive ACR/EULAR-aPL in the absence of documented autoimmune disorders, have been demonstrated to increase the risk of thromboembolic events, including IS, transient ischemic attack (TIA), VTE, and recurrent miscarriages [18].

ANA and ACR/EULAR-aPL frequently coexist, with ANA positivity varying depending on the specific type of ACR/EULAR-aPL present: 29.2% in those with lupus anticoagulant (LA), 16.7% with positive anti-β2 glycoprotein I (anti-β2GPI), and 12.5% with positive anticardiolipin antibodies (aCL) [19]. Notably, double- and triple-positive ACR/EULAR-aPL profiles show higher ANA rates, at 57% and 41.7%, respectively [19]. However, different ACR/EULAR-aPL isotypes influence thromboembolic risk differently, data on their coexistence with ANA and the associated risk remain limited.

Recently, we have reported a relatively high (21%) prevalence of positive ACR/EULAR-aPL at moderate or high titers in AF patients treated with direct oral anticoagulants (DOACs) and positive ACR/EULAR-aPL were associated with an increased long-term risk of IS [20]. We hypothesized that increased prevalence of positive ANA may contribute to thromboembolic and death risks in AF patients on anticoagulation. The aim of this study was to investigate whether positive ANA, with or without positive ACR/EULAR-aPL, in elderly AF patients on anticoagulation is associated with clinical outcomes during follow-up.

## Materials and methods

### Patients

We determined ANA in blood samples obtained from 240 adult patients with AF between June 2014 and July 2016. The study population has been presented in detail previously [20], however, ANA levels were not available in 3 patients from the previous cohort. Briefly, AF was diagnosed according to ESC guidelines [21]. The exclusion criteria were: recent arterial or venous thromboembolic events, advanced heart failure chronic kidney disease, liver injury, malignancy, acute infection, and C-reactive protein (CRP) > 10 mg/L. Patients with known autoimmune diseases were not eligible. The study received approval from the Jagiellonian University Ethics Committee, and all participants provided written informed consent.

Thromboembolic risk in AF patients was assessed using the CHA2DS2-VASc score, and the high risk category was defined as ≥ 2 for men and ≥ 3 for women [1]. Ischemic stroke was diagnosed based on clinical symptoms and positive findings on imaging (CT or MRI). Patient characteristics, such as hypercholesterolemia, hypertension, diabetes, BMI, and heart failure, were defined as previously described [20].

### Laboratory investigation

In patients on DOAC, in whom the time since the last dose was below 24 h, the DOAC-Stop (Haematex Research, Sydney, Australia) was used to remove DOAC from plasma [22]. In patients treated with warfarin, blood samples were drawn 24 h after the last dose of low molecular weight heparin intake. Routine laboratory tests were used to assess blood cell count, glucose, creatinine, lipid profile, plasminogen, and antiplasmin. High-sensitivity CRP was evaluated by nephelometry (Siemens, Marburg, Germany). Von Willebrand factor antigen levels were determined using a latex immunoassay (Diagnostica Stago, Asnières, France). Plasminogen activator inhibitor-1 (PAI-1) and thrombin-activatable fibrinolysis inhibitor (TAFI) antigens were measured by ELISA (Hyphen-Biomed, Neuville-Sur-Oise, France).

### Antinuclear antibodies

ANA levels were measured using the Quanta Lite™ ELISA test (Inova Diagnostics, San Diego, CA) and were expressed as ELISA units (EUANA levels exceeding 1 EU were considered detectable, and levels above 20 EU were classified as'positive,'according the manufacturer [23].

### Antiphospholipid antibody

As described previously [21], LA was detected using clot-based assays, and aCL and anti-β2GPI antibodies were measured by ELISA (INOVA Diagnostics, San Diego, CA, US). Positive results for anti-β2GPI and aCL antibodies were defined as titers of ≥ 40 units for both IgG and IgM isotypes. Double ACR/EULAR-aPL positivity was defined as the presence of any two of the following antibodies: aCL, or anti-β2GPI, and triple ACR/EULAR-aPL positivity was defined as the presence of two antibodies with positive LA [24]. For both double and triple positivity, aCL and anti-β2GPI had to be in the same isotype.

### Follow-up

Follow-up was conducted by phone calls or through visits at least twice a year. The primary endpoints were IS or TIA diagnosed based on World Health Organization Criteria [25]. The secondary endpoint were major bleeding, defined according to the International Society on Thrombosis and Hemostasis (fatal bleeding or symptomatic bleeding in a critical area or organ, or bleeding accompanied by a decrease in the hemoglobin level of ≥ 2 g/dL or leading to transfusion of ≥ 2 U of whole blood/packed red blood cells) [26]. Composite endpoint was defined as occurrence of IS or TIA or CV death during follow-up.

### Statistical analysis

Based on available data, the minimum number of individuals with clinical outcomes in AF patients treated with DOACs was determined to be 25 [27]. Continuous variables were reported as either mean ± standard deviation (SD) or median with interquartile range (IQR). The Shapiro–Wilk test was used to check for normality of the data. Categorical variables were expressed as counts and percentages, and compared using either Pearson’s χ^2^ test or Fisher’s exact test. For comparing two groups, the Student’s t-test was applied for normally distributed continuous variables, while the Mann–Whitney U test was used for non-normally distributed ones. In cases involving multiple groups, ANOVA was used for normally distributed variables, and the Kruskal–Wallis test for non-normally distributed ones. Correlation between variables was assessed using Spearman’s test for non-normal distributions and Pearson’s test for normal distributions. Logistic regression analysis was performed to assess whether ANA can predict the composite endpoint and results were shown as odds ratio (OR) and 95% confidence interval (CI).

All statistical analyses were carried out using STATISTICA 13 (StatSoft, Tulsa, OK, US), Python (SciPy 1.11.3, Austin, TX, US), and R libraries 4.1.1 (The R Foundation for Statistical Computing, Vienna, Austria, 2021).

## Results

### Baseline characteristics

Among 240 consecutive AF patients (44.6% women), at a median age of 69 years (63–75) (Table 1), the majority of patients were at high risk for thromboembolism (*n* = 221; 92.1%) at a median CHA2DS2-VASc score of 4 (3–5). In terms of anticoagulation therapy, 80 (33.3%) patients were on rivaroxaban, 56 (23.3%) on dabigatran, 35 (14.6%) on apixaban, and 61 (25.4%) on warfarin. Only 11 patients (4.6%) reported an interruption of anticoagulation, primarily for less than one month, at their last follow-up.

**Table 1:** Baseline characteristics based on the presence of antinuclear antibodies and antiphospholipid status

Variable	All (*n* = 240)	Positive ANA (> 20 EU) *n* = 43	Negative ANA (≤ 20 EU) *n* = 197	*P* value	Positive ANA with ACR/EULAR-aPL (*n* = 20)	Positive ANA without ACR/EULAR-aPL (*n* = 23)	*P* value
Age, years	69 (63–75)	77 (73–80)	68 (62–73)	< 0.001	74.5 (70.5–79.5)	77 (76–80)	0.14
Women, n (%)	107 (44.6)	28 (65.12)	79 (40.1)	< 0.001	14 (70)	14 (60.87)	0.53
Body-mass index, kg/m^2^	28.1 (25.4–31.6)	27.4 (25.6–30.9)	28.3 (25.4–31.7)	0.73	27.1 (25.5–29.0)	28.2 (26.0–32.5)	0.32
Time from AF diagnosis, years	6 (4–8.5)	6 (5–12)	6 (4–8)	0.3	6 (5–9)	8 (3–15)	0.41
CHA2DS2-VASc	4 (3–5)	5 (4–6)	4 (3–5)	< 0.001	5 (4–6)	5 (4–6)	0.37
**Comorbidities and cardiovascular disease risk factors, n (%)**							
Smoking	87 (36.3)	13 (30.23)	74 (37.56)	0.4	7 (35)	6 (26.09)	0.31
Hypertension	187 (77.91)	34 (79.07)	153 (77.66)	0.72	14 (70)	20 (86.96)	0.17
Diabetes mellitus	71 (29.58)	13 (30.23)	58 (29.44)	0.83	8 (40)	4 (17.39)	0.1
Myocardial infarction	71 (29.58)	13 (30.23)	58 (29.44)	0.87	8 (40)	5 (21.74)	0.19
HFrEF	57 (23.75)	10 (23.26)	47 (23.86)	0.76	6 (30)	4 (17.39)	0.33
Arterial disease	120 (50)	23 (53.49)	97 (49.24)	0.55	11 (55)	12 (52.17)	0.85
Prior stroke/TIA/TE	97 (40.42)	19 (44.19)	78 (39.59)	0.53	12 (60)	7 (30.43)	0.051
**Medications, n (%)**							
Acetylsalicylic acid	105 (43.75)	17 (39.53)	88 (44.67)	0.59	8 (40)	9 (39.13)	0.95
Statin	157 (65.42)	31 (72.09)	126 (63.96)	0.26	15 (75)	16 (69.57)	0.69
ACEI	127 (52.91)	29 (56.87)	98 (49.75)	0.4	11 (55)	14 (60.87)	0.7
Angiotensin receptor blocker	58 (24.17)	25 (58.14)	33 (16.75)	0.62	3 (15)	6 (26.09)	0.37
Diuretic	104 (43.33)	9 (20.93)	95 (48.22)	0.4	5 (25)	11 (47.83)	0.12
Beta-blocker	23 (9.58)	3 (6.98)	20 (10.15)	0.54	3 (15)	0 (0)	0.054
Amiodaron	26 (10.83)	8 (18.6)	18 (9.14)	0.06	5 (25)	3 (13.04)	0.31
Rivaroxaban	80 (33.33)	14 (32.56)	66 (33.5)	0.96	9 (45)	5 (21.74)	0.1
Dabigatran	56 (23.33)	10 (23.26)	46 (23.35)	0.97	4 (20)	6 (26.09)	0.64
Apixaban	35 (14.58)	7 (16.28)	28 (14.21)	0.7	2 (10)	5 (21.74)	0.3
Warfarin	61 (25.42)	12 (27.91)	49 (24.87)	0.64	5 (25)	7 (30.43)	0.69
**Laboratory investigations**							
White blood cells, × 10^3^/μL	6.7 (5.53–7.56)	6.29 (5.3–7.56)	6.74 (5.63–7.62)	0.32	6.16 (5.42–7.67)	6.3 (5.15–7.29)	0.99
Platelet count, × 10^3^/μL	210 (176–251)	211 (163–238)	208 (178.5–252)	0.65	217.5 (173–246)	210 (155–234)	0.58
Glucose, mmol/L	5.45 (4.9–6.2)	5.3 (4.9–6.1)	5.5 (4.9–6.21)	0.61	5.06 (4.9–6)	5.4 (4.9–6.1)	0.54
Creatinine, μmol/L	82 (71.7–98.5)	84 (72–110.2)	82 (71.7–98.25)	0.41	87 (70.5–120)	83.2 (73.8–97)	0.75
LDL cholesterol, mmol/L	2.59 (2.03–3.4)	2.43 (2.04–3.33)	2.6 (2.02–3.4)	0.65	2.42 (2.01–3.11)	2.47 (2.1–3.47)	0.52
NT-proBNP, pg/mL	748 (391–1493)	805 (419–1581)	745 (388–1491)	0.76	765 (356–2667)	976 (463–1168)	0.93
**Coagulation and fibrinolysis parameters**							
Fibrinogen, g/L	3.2 (2.52–3.92)	3.34 (2.53–3.8)	3.15 (2.51–3.97)	0.85	3.42 (2.72–3.84)	3.19 (2.35–3.74)	0.28
TAFI antigen, %	100 (89–110)	102 (95–117)	99 (89–108)	0.02	101 (90.5–114)	102 (99–119)	0.24
Plasminogen, %	105 (95–115)	104 (92–115)	105 (95–116)	0.44	100 (89–111.5)	109 (98–117)	0.29
PAI-1 antigen, ng/mL	14 (10.8–18.7)	14 (11.5–20)	14.1 (10.6–18.7)	0.88	13.7 (11.5–17.8)	15.2 (11.4–18.8)	0.67
vWF, %	208 (173–243)	197 (151–243)	210 (176–243)	0.16	209.65 ± 53.03	288.57 ± 44.58	0.23
Antiplasmin, %	107 (96–117)	107 (99–116)	106 (96–117)	0.81	107.5 (100–118)	107 (99–116)	0.49
**Follow-up**							
IS/TIA, n (%)	20 (8.33)	6 (13.95)	14 (7.11)	0.13	4 (20)	2 (8.7)	0.29
Major bleeding, n (%)	23 (9.58)	5 (11.63)	18 (9.14)	0.73	2 (10)	3 (14.29)	0.94
Cardiovascular death, n (%)	12 (5)	6 (13.95)	6 (3.05)	< 0.001	4 (20)	2 (8.7)	0.29
Death, n (%)	20 (8.33)	10 (23.26)	10 (5.08)	< 0.001	6 (30)	4 (17.39)	0.33

Data are presented as median (interquartile range), number (percentage) or mean standard deviation. Abbreviations: *AF* atrial fibrillation, NT-proBNP N-terminal pro-B-type natriuretic peptide, *n* number, *LDL* low-density lipoprotein, K_s_ clot permeability, *CLT* clot lysis time, *PAI-1* plasminogen activator inhibitor-1, *TAFI* thrombin-activatable fibrinolysis inhibitor, vWF von Willebrand factor activity, *TIA* transient ischemic attack, *ANA* antinuclear antibodies

**Fig. 1:**
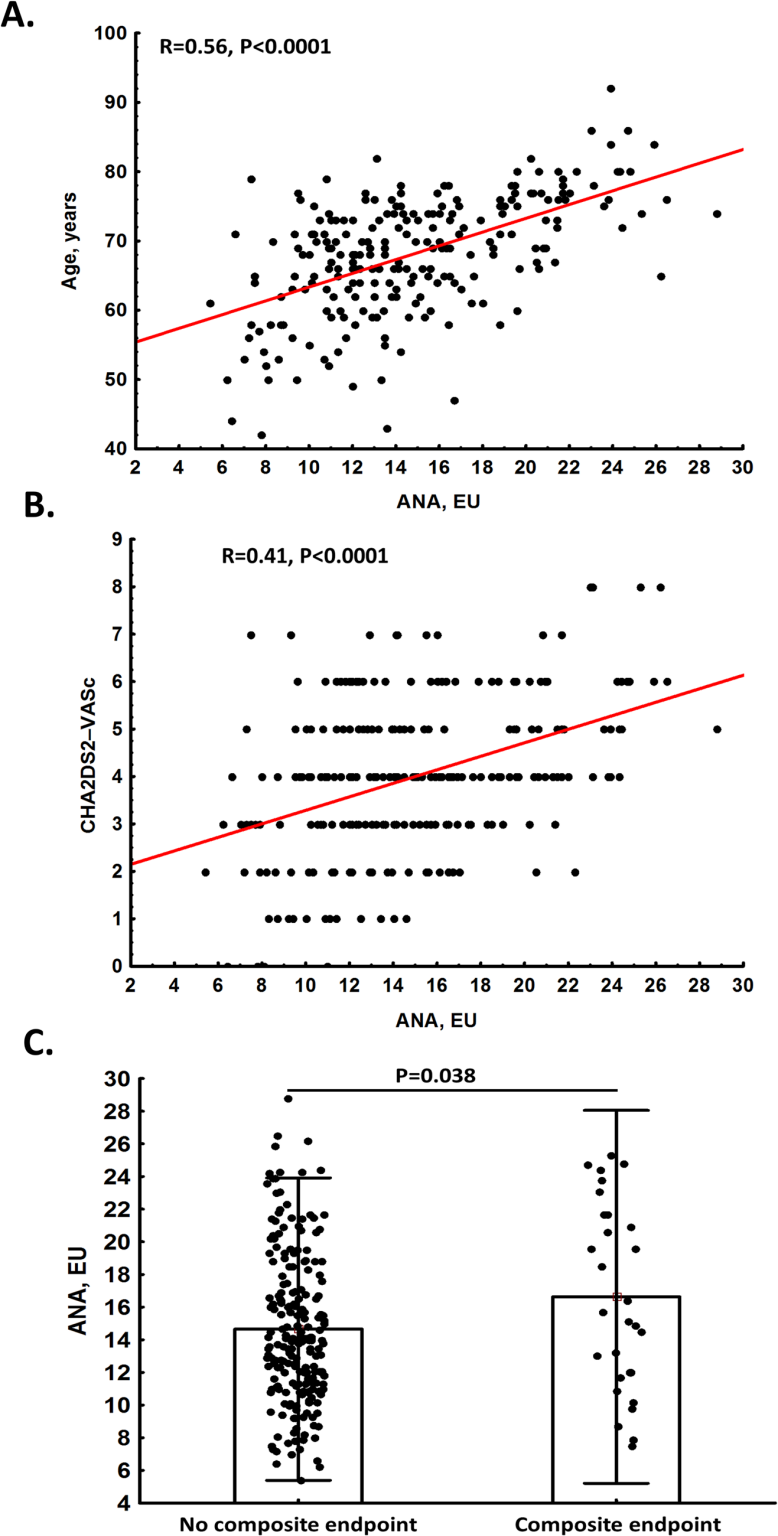
Associations of ANA levels with age (panel **A**), CHA2DS2-VASc score (panel **B**, and the composite endpoint occurrence (panel **C**)

Measurable ANA were detected in all patients (14.1 [11.3–18.5] EU), including positive ANA in 43 subjects (17.9%) with a median concentration of 21.8 [IQR 20.9–24.3] EU and a maximum value of 28.8 EU. None of the patients met criteria for autoimmune diseases other than antiphospholipid syndrome (*n* = 17, 7.1%). ANA positive patients were older by 9.9 years with overrepresentation of females (Table 1). They also had higher CHA2DS2-VASc scores (Table 1). However, when age was removed from the CHA2DS2-VASc scores, the difference between ANA positive and negative patients became non-significant (*P* > 0.05). ANA levels showed positive correlations with age (Fig. 1A) and CHA2DS2-VASc scores (Fig. 1B), that remained significant even when age was excluded from the score (r = 0.18, *P* < 0.001). No significant differences were observed between the ANA positive and ANA negative groups regarding comorbidities and medications (Table 1). Analysis of laboratory investigations showed no ANA-related differences with one exception, namely TAFI antigen levels were slightly higher in the ANA positive group (Table 1).

Among 43 ANA positive patients, 20 (46.51%) had also positive ACR/EULAR-aPL, including 13 (30.23%) single ACR/EULAR-aPL (LA, *n* = 11 [25.58%]; aCL IgM *n* = 1 [2.33%]; anti-β2GPI IgG n = 1 [2.33%]), 2 (4.65%) double ACR/EULAR-aPL (LA + aCL IgM *n* = 1 [2.33%]; anti-β2GPI IgM + aCL IgM *n* = 1 [2.33%]) and 5 (11.6%) triple ACR/EULAR-aPL (LA + anti-β2GPI IgM + aCL IgG *n* = 1 [2.33%]; LA + anti-β2GPI IgM + aCL IgG *n* = 2 [4.65%]; LA + anti-β2GPI IgM and IgG + aCL IgM and IgG *n* = 2 [4.65%]) (Table 3). Comparison of ANA positive patients with ACR/EULAR-aPL versus without ACR/EULAR-aPL revealed no significant differences (Table 1).

### Follow up

During a median follow-up of 52 months (46–56), we observed composite endpoint in 30 (12.5%) patients who had median ANA levels of 15.7 (IQR 12–21.7) EU (min 7.5 and max 25.3 EU), including 10 (33.3%) ANA positive cases, of whom 7 (70%) had also positive ACR/EULAR-aPL (Table 2). There were 20 episodes of IS in patients with median ANA value of 14.8 (IQR 10.6–21.9) EU, including 6 ANA positive cases (20%) with median value of 24.6 (IQR 23.1–24.8) EU, among whom 4 cases (66.7%) were positive for both ANA and ACR/EULAR-aPL with median value of 24.8 (IQR 24.7–25.3) EU (Table 1). As shown in Table 1, a total of 20 deaths were recorded. Of these, 10 deaths (50% of all recorded deaths) occurred in the ANA positive group, with median ANA value of 21.7 (IQR 20.8–24.2) EU, including 4 (40% of all recorded deaths in the ANA positive group) in patients positive for both ANA and ACR/EULAR-aPL (median ANA value of 21.3 [IQR 20.9–23.2] EU). We observed 6 (50%) CV deaths, among ANA positive patients, including 4 (66.7%) positive for both ANA and ACR/EULAR-aPL and 2 (33.3%) positive solely for ANA (Table 1). Furthermore, 23 (9.6%) major bleeding episodes were observed, with 5 (21.7%) cases in ANA positive patients, including 2 (40%) cases positive for both ANA and ACR/EULAR-aPL (Table 1).

**Table 2:** Antibody profile based on the presence or absence of composite endpoint during follow-up, defined as ischemic stroke, cardiovascular death, or all-cause mortality

Variable	All (*n* = 240)	Composite endpoint (*n* = 30)	No composite endpoint (*n* = 210)	*P* value
Positive ACR/EULAR-aPL, n (%)	51 (21.25)	16 (53.33)	35 (16.67)	< 0.01
Positive LA, n (%)	37 (15.42)	8 (26.67)	29 (13.81)	0.063
Positive anti-β2GPI IgG, n (%)	9 (3.75)	2 (6.67)	7 (3.33)	0.36
Positive anti-β2GPI IgM, n (%)	9 (3.75)	4 (13.33)	5 (2.38)	< 0.01
Positive aCL IgG, n (%)	9 (3.75)	3 (10)	6 (2.89)	0.051
Positive aCL IgM, n (%)	13 (5.42)	4 (13.33)	9 (4.29)	< 0.01
Positive ANA, n (%)	43 (17.92)	10 (33.33)	33 (15.71)	0.017
Positive ANA with ACR/EULAR-aPL, n (%)	20 (8.33)	7 (23.33)	13 (6.19)	< 0.01
CHA2DS2-VASc	4 (3–5)	4 (4–6)	4 (3–5)	0.046

Data are presented as number (percentage). Abbreviations: *ACR/EULAR-aPL* antiphospholipid antibodies in accordance to ACR/EULAR, *LA* lupus anticoagulant, *anti-β2GPI* anti-beta-2 glycoprotein I antibody, *IgG* immunoglobulin G, *IgM* immunoglobulin M, *aCL* anticardiolipin antibody, *ANA* antinuclear antibodies

**Fig. 2:**
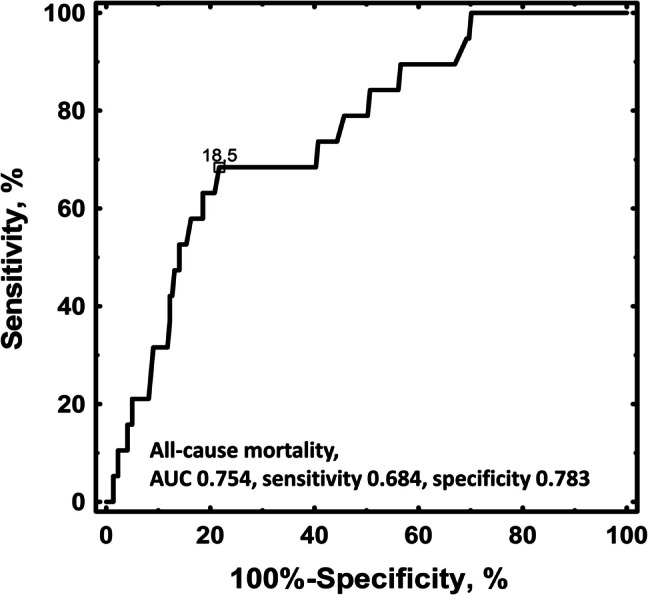
ROC analysis of ANA levels and their association with the incidence of all-cause mortality

The ROC curve analysis for ANA levels indicated an optimal cut-off value of 18.50 EU for predicting all-cause mortality (Fig. 2). No differences in IS and major bleeding, were observed between ANA positive and ANA negative groups, regardless of co-existence of ACR/EULAR-aPL (Table 1). However, all-cause mortality and CV death occurred more frequently in ANA positive patients (Table 1). The composite endpoint was more frequently observed in patients with positive ANA, positive ACR/EULAR-aPL, or those with both ANA- and ACR/EULAR-aPL -positive (Table 2). However, when considering ANA positivity without ACR/EULAR-aPL, no differences in the rate of the composite endpoint were found (*P* = 0.2). Patients who experienced the composite endpoint during follow-up generally had 18% higher ANA levels compared to the remainder (Fig. 1C).

On univariable regression analysis ANA positivity was associated with the occurrence of composite endpoint (OR = 2.84, 95% CI: 1.21–6.65, *P* = 0.016), even after adjustment for age (OR = 2.82, 95% CI: 1.03–7.73, *P* = 0.045). Importantly, combined ANA and ACR/EULAR-aPL positivity markedly increased the odds for composite endpoint occurrence (OR = 4.85, 95% CI 1.75–13.43, *P* = 0.002) Table 3.

**Table 3:** Distribution of ACR/EULAR-aPL among ANA positive patients

Variable	Positive ANA (> 20 EU) n = 43
Positive ACR/EULAR-aPL, n (%)	20 (46.52)
Positive LA, n (%)	16 (37.21)
Positive anti-β2GPI IgG, n (%)	5 (11.63)
Positive anti-β2GPI IgM, n (%)	5 (11.63)
Positive aCL IgG, n (%)	5 (11.63)
Positive aCL IgM, n (%)	5 (11.63)

Data are presented as number (percentage). Abbreviations: *ACR/EULAR-aPL* antiphospholipid antibodies in accordance to ACR/EULAR, *LA* lupus anticoagulant, *anti-β2GPI* anti-beta-2 glycoprotein I antibody, *IgG* immunoglobulin G, *IgM* immunoglobulin M, *aCL* anticardiolipin antibody, *ANA* antinuclear antibodies

## Discussion

To the best of our knowledge, this is the first study to evaluate the prevalence and prognostic significance of ANA in a cohort of patients with AF while on anticoagulation. Our findings indicate that ANA positivity is relatively common among AF patients (17.9%) and is associated with an increased thromboembolic risk during long-term follow-up. Our findings indicate that AF patients with both ANA positivity and coexisting ACR/EULAR-aPL are at a greater risk of thromboembolism and cardiovascular death compared to those with ANA positivity alone. The current study provides the first evidence indicating that complex autoimmune mechanisms increasingly prevalent among older individuals might contribute to the failure of DOAC anticoagulation in AF, which suggests that ANA evaluation might help identify subjects more prone to adverse events while on anticoagulation. It is tempting to speculate that interventions aimed at decreasing ANA titers might be beneficial in such a subset of AF patients especially those with prior cerebrovascular ischemic events.

The prevalence of positive ANA in our AF patients was 17.9%, placing it within the higher range reported in the general population, which reaches up to 30% [5]. This may partly be attributed to the association of AF with older age or the elevated inflammatory status linked to AF [28, 29]. However, a study by Hu et al. [16] comparing ANA prevalence in a healthy, age-matched control group and AF patients found no statistically significant differences, suggesting that age may be the primary contributing factor.

Interestingly, in the current study, 46.5% of ANA positive patients also had positive ACR/EULAR-aPL. This is a notably high percentage, especially given that positive ACR/EULAR-aPL are typically found in only 30% to 40% of SLE patients, a condition in which ANA positivity approaches 99% [30]. In our research, ANA positivity was associated with both cardiovascular death and overall mortality, also after adjustment for age, which is consistent with findings from other studies [31]. Patients positive for both ANA and ACR/EULAR-aPL had nearly twice the odds of reaching the composite endpoint compared to those with ANA positivity alone. However, ANA positivity alone was not associated with an increased likelihood of the composite outcome. This suggests that while ANA may play a role in thromboembolic risk, its impact is significantly amplified when ACR/EULAR-aPL are also present. This highlights the potential for a stronger combined effect, though further research is needed to fully elucidate this association.

Aging is known to affect immune markers, including ANA [8]. This age-related trend likely explains the higher CHA2DS2-VASc scores observed in ANA positive patients. Notably, none of the other common CV death risk factors, such as hypercholesterolemia or diabetes, were associated with increased ANA positivity. Our findings suggest that the age-related increase in ANA prevalence, along with the associated thromboembolic risk, may play a more significant role in patient outcomes than previously recognized.

The mechanisms connecting ANA positivity to increased mortality or heightened thromboembolic risk remain unclear. One possibility is that pre-existing cardiovascular conditions like AF may cause the release of intracellular antigens, triggering ANA production [28]. This process, combined with an elevated inflammatory status, could contribute to increased ACR/EULAR-aPL levels and, through this mechanism, heighten the thromboembolic risk in ANA-positive AF patients [29, 32]. Alternatively, ANA may contribute directly to increased CV death or IS in AF patients by inducing endothelial dysfunction and damage [11, 33, 34]. This process could accelerate atherosclerosis, further amplifying the risk beyond that attributed to ACR/EULAR-aPL alone [11, 32, 34]. A study of 103 ANA positive patients identified small high-intensity spots on brain MRI as the most common finding [35], aligning with Yang et al. [36] findings that ACR/EULAR-aPL -associated strokes primarily affect smaller arteries, supporting the potential link between ANA complications and ACR/EULAR-aPL. It is also worth noting that emerging evidence suggests autoimmune responses, including the formation of specific autoantibodies like anti-potassium channel Kir3.4, may contribute to the development and progression of AF, which further supports the potential role of various autoantibodies in the pathogenesis and prognosis of AF [37].

There is also the question of whether lower ANA levels contribute to increased thromboembolic risk or if they must cross the threshold of ANA positivity to have a significant impact. Even low ANA levels are associated with an increased thromboembolic risk; however, this risk rises logarithmically, with higher ANA levels significantly amplifying the risk compared to lower titers [9]. However, Blann et al. [38] observed no significant correlation between ANA titers and vascular endothelial damage, suggesting that the mere presence of ANA may be the more critical factor influencing thromboembolic risk. Further studies are needed to determine which threshold of ANA levels is more critical for thromboembolic risk and to identify the most beneficial threshold for guiding clinical decisions in patients with AF.

This study has several limitations. The sample size, particularly the number of patients with positive ANA without ACR/EULAR-aPL, was relatively small. However, the high coexistence rate of ANA and ACR/EULAR-aPL observed in our cohort suggests a strong likelihood of their concurrent presence in AF patients. Another limitation of this study is that ANA levels were measured using ELISA, therefore our results cannot be directly compared with the results of some studies, where ANA were assessed by immunofluorescence [39]. Moreover, we did not differentiate between specific ANA patterns (e.g., speckled, homogeneous, centromere, cytoplasmic), which can influence thromboembolic risk differently [40, 41]. Additionally, we did not distinguish between ANA subtypes, such as anti-dsDNA, which is specifically associated with systemic lupus erythematosus [42]. However, our focus was on a more accessible and cost-effective general ANA measurement. While this may limit the detailed understanding of their specific impact on overall risk, assessing these patterns was beyond the scope of this study. A significant limitation of this study is the lack of existing data and basic science research on ANA in AF patients and its association with increased mortality. This gap in knowledge limits the ability to fully understand the underlying mechanisms and clinical implications of ANA positivity in this population. Additionally, ANA levels were measured only once, and we cannot exclude that after a few years ANA levels increased and some patients negative for ANA at baseline became positive or some with low ANA titers became negative. Prospective studies with repeated measurements of ANA are needed to provide a more comprehensive understanding of their role in thromboembolic risk in AF patients.

## Conclusion

Our findings demonstrated that elevated ANA levels may contribute to increased risk of adverse cardiovascular events in AF patients potentially explaining, at least in part, the failure of anticoagulant prevention—an issue unlikely to be resolved by current or emerging anticoagulant therapies. Future large studies are needed to assess the feasibility and usefulness of ANA testing in AF patients for improved thromboembolic risk stratification.

## Data Availability

The data supporting this study are available from the corresponding author upon request.
